# PPARα agonist and metformin co-treatment ameliorates NASH in mice induced by a choline-deficient, amino acid-defined diet with 45% fat

**DOI:** 10.1038/s41598-020-75805-z

**Published:** 2020-11-11

**Authors:** Shinya Okishio, Kanji Yamaguchi, Hiroshi Ishiba, Nozomi Tochiki, Kota Yano, Aya Takahashi, Seita Kataoka, Keiichiroh Okuda, Yuya Seko, Yu Liu, Hideki Fujii, Daiki Takahashi, Yusuke Ito, Junji Kamon, Atsushi Umemura, Michihisa Moriguchi, Kohichiroh Yasui, Takeshi Okanoue, Yoshito Itoh

**Affiliations:** 1 Molecular Gastroenterology and Hepatology, Graduate School of Medical Science, Kyoto Prefectural University of Medicine, 465 Kajii-cho, Kawaramachi-Hirokoji, Kamigyou-ku, Kyoto, 602-8566 Japan; 2 Pharmaceutical Research Department, Biological Research Laboratories, Nissan Chemical Corporation, Saitama, Japan; 3 Department of Gastroenterology and Hepatology, Saiseikai Suita Hospital, Osaka, Japan

**Keywords:** Non-alcoholic fatty liver disease, Non-alcoholic steatohepatitis

## Abstract

We explored the beneficial effects of GW7647, a peroxisome proliferator activated receptor α (PPARα) agonist, and metformin, an anti-diabetic drug on an advanced nonalcoholic steatohepatitis (NASH) model in rodents and investigated the possible mechanisms involved. Mice were fed control chow or a choline-deficient l-amino acid-defined diet containing 45% fat (HF-CDAA). The mice fed HF-CDAA diets for 16 weeks were divided into four groups: the no treatment (HF-CDAA), HF-CDAA containing 1000 mg/kg metformin, HF-CDAA containing 10 mg/kg GW7647, and HF-CDAA with both metformin and GW7647 groups. Metformin alone slightly deteriorated the aspartate and alanine aminotransferase (AST/ALT) values, whereas co-treatment with GW7647 and metformin greatly suppressed liver injury and fibrosis via activation of the AMP-activated protein kinase (AMPK) pathway. Further study revealed that co-treatment decreased the expression of inflammatory-, fibrogenesis-, and endoplasmic reticulum (ER) stress-related genes and increased the oxidized nicotinamide adenine dinucleotide (NAD)/reduced nicotinamide adenine dinucleotide (NADH) ratio, suggesting the superiority of co-treatment due to restoration of mitochondrial function. The additive benefits of a PPARα agonist and metformin in a HF-CDAA diet-induced advanced NASH model was firstly demonstrated, possibly through restoration of mitochondrial function and AMPK activation, which finally resulted in suppression of hepatic inflammation, ER stress, then, fibrosis.

## Introduction

Nonalcoholic fatty liver disease (NAFLD) is one of the most common liver diseases and is significantly associated with features of metabolic syndrome, including obesity, dyslipidemia, and insulin resistance^1,2^. While liver fibrosis severity predicts mortality and time to development of severe liver disease in NAFLD, emerging evidence indicates that metabolic inflammation is a key process in the progression of simple steatosis to more advanced stages of liver damage and fibrosis^3,4^. Several inflammatory pathways and mediators have been implicated to associate with NASH, leading to occurrence of type 2 diabetes mellitus, cardiovascular disease, and chronic kidney disease^5–7^. However, the pathogenesis of NAFLD is multifactorial with limited pharmacotherapeutic options for the treatment of patients^8,9^.

PPARs are not only drug targets of glucose and lipid metabolism, but they can also be used to treat other diseases, such as primary biliary cholangitis^10^. PPARs are nuclear receptors that play key roles in cellular processes that control lipid and carbohydrate metabolism in the liver, muscle, and adipose tissues^11^. PPARα is most prominently expressed in the liver, where it serves as the master transcriptional regulator of hepatic fatty acid transport and β-oxidation^11,12^. In addition, PPARα activation inhibits inflammation-related genes induced by nuclear factor-κB (NF-κB) and decreases the expression of acute-phase response genes in a peroxisome proliferator response element (PPRE)-dependent or PPRE-independent manner^12–14^. Hence, PPARα agonists are now also being investigated as potential anti-NASH drugs and are currently under evaluation in clinical trials^15^.

Metformin is an also commonly used medication for type 2 diabetes mellitus, as some evidence suggests beneficial effects in hepatocellular carcinoma and other cancers^16,17^. In hepatocytes, metformin increases the AMP/ATP ratio and AMPK activity via inhibition of the mitochondrial respiratory chain (complex I) and decreases gluconeogenesis and increases fatty acid oxidation, which supports its use in NAFLD^18^. AMPK is a key enzyme that participates in insulin signaling, whole-body energy balance, and the metabolism of glucose and fats^19,20^. Furthermore, several studies have demonstrated that metformin suppressed inflammatory responses through inhibition of c-Jun N-terminal kinase (JNK) and NF-κB in various types of cells^21,22^. Although the precise underlying mechanism remains unclear, it was recently reported that AMPK signals inhibit the activation of caspase-6 via phosphorylation of procaspase-6 and the consequent caspase activation cascade, which leads to apoptosis in various murine NASH models^23^. Metformin has also been shown to exhibit anti-fibrotic effects independent of its lipid-lowering activity in several organs^24,25^. However, not all individuals with chronic liver disease with impaired mitochondrial function who are prescribed metformin derive the same benefits, and some even develop adverse effects^26,27^.

Previously, only a few studies demonstrated the potential benefits of tesaglitazar, a dual PPARα/γ agonist, as an add-on therapy to metformin in patients with poorly controlled type 2 diabetes mellitus^28^. The overlapping pathways controlled by PPARs and AMPK prompted us to investigate whether their combined agonist activities could promote additive benefits for the metabolic and histological outcomes in NASH. To address this question, the present study assessed the therapeutic efficacy of treatment with a PPARα agonist and/or metformin using a recently described mouse model of NASH. Feeding rodents a CDAA diet containing high-fat components has been reported to cause excess oxidative/ER stress in the liver and to induce liver steatosis, inflammation, and fibrosis within 12–16 weeks^29^. This useful animal model has provided us with the opportunity to assess the pathophysiology of progressive NAFLD in humans.

We investigated the anti-inflammatory, anti-oxidant, and anti-fibrogenic roles of the PPARα agonist GW7647, and metformin. Rather than protecting the liver from the development of NASH, metformin was harmful and worsened the serum AST and ALT value in this mouse model of advanced NASH. On the contrary, GW7647 enhanced hepatic expression of β-oxidation-related genes and reduced serum triglyceride (TG) levels. Particularly, co-treatment with GW7647 and metformin consistently improved the serum AST and ALT value, liver steatosis, liver injury, and fibrosis with a recovery of insulin sensitivity in the liver. Interestingly, improvement in progressive liver damage by this co-treatment occurred despite a decrease in hepatic fibroblast growth factor 21 (FGF21) expression and serum FGF21 levels, which suggests amelioration of hepatic ER stress and FGF21 resistance induced by HF-CDAA^30^. These findings suggest that in this model, co-treatment with a PPARα agonist and metformin provides a useful mechanism to protect the liver from metabolic inflammation.

## Results

### HF-CDAA-induced progressive NASH in mice

**Figure 1:**
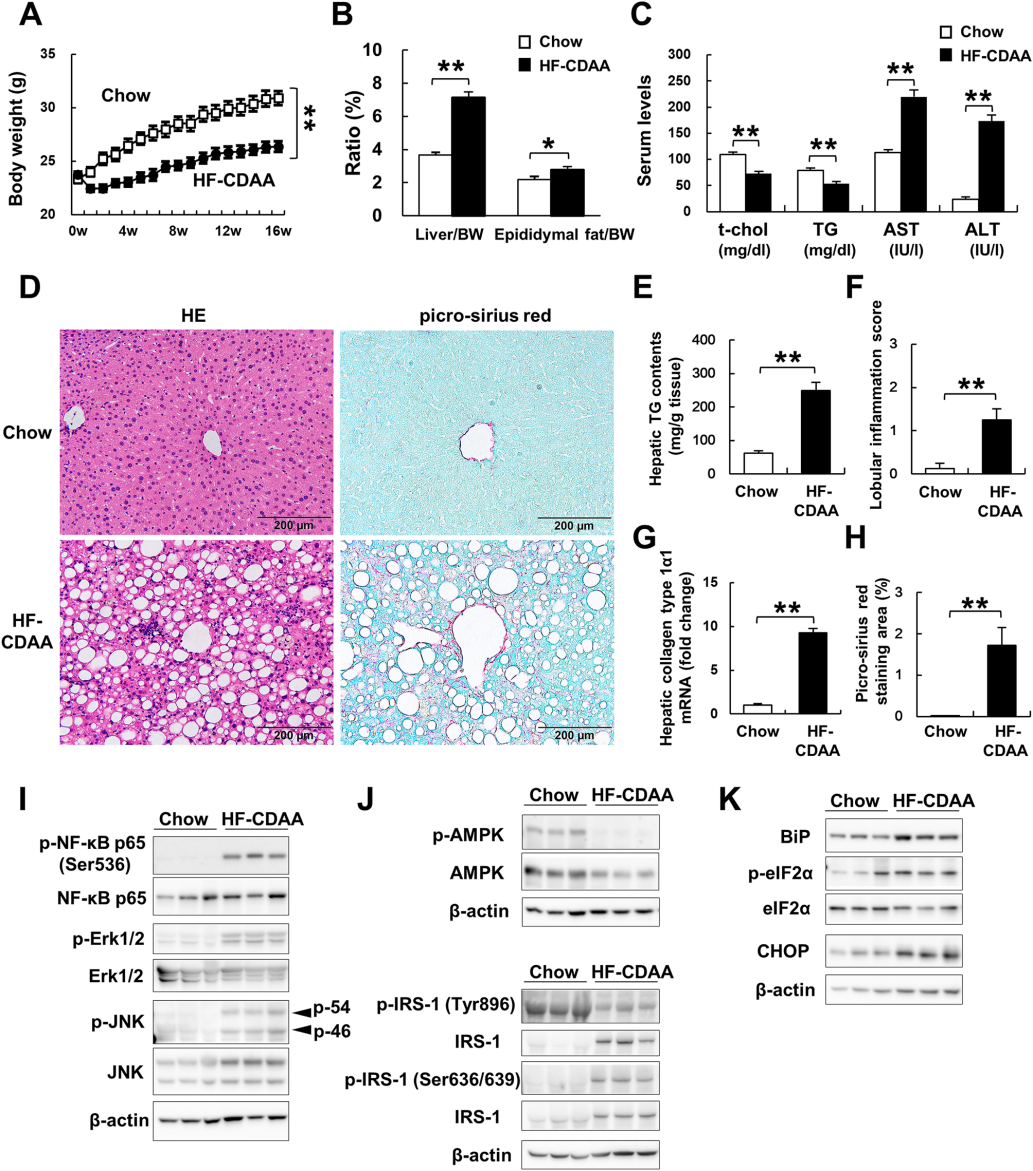
HF-CDAA diets-induced NASH. (**A**) Body weight changes during 16-week treatment are plotted. (**B**) Liver/body weight (BW) and epididymal fat/BW ratio (%) were assessed at 16 weeks. (**C**) Serum total cholesterol, triglyceride, AST and ALT values were determined. (**D**) Hematoxylin and eosin, and picro-sirius red staining of liver sections from representative mice fed chow or HF-CDAA diets. (**E**) Hepatic TG contents were measured. (**F**) The number of inflammatory foci per 200 × field was counted on sections from each mouse. Mean ± SE data from each group (n = 8/group) are displayed. (**G**) Hepatic mRNA levels of type1 α1 procollagen were determined by quantitative real time PCR analysis. Results were normalized to glucuronidase expression. Mean ± SE data are displayed as fold changes relative to chow-fed mice. (**H**) Morphometric analysis of picro-sirius red-stained sections from each group (n = 8/group). Results are expressed as percentage of section staining (+) for picro-sirius red. (**I**) Phosphorylated (p)-NF-κB p65 at ser536, total (t)-NF-κB p65, p-Erk1/2 at Thr202/Tyr204, t-Erk1/2, p-JNK at Thr183/Tyr185 and t-JNK, (**J**) p-AMPKα at Thr172, t-AMPKα, p-IRS-1 at Tyr896 and at Ser636/639, t-IRS-1, (K) p-eIF2α at Ser51, t-eIF2α and CHOP were evaluated by immunoblot analysis of livers from 3 mice/group. To control for loading, the blot was stripped and re-probed for actin. *p < 0.05, **p < 0.01. *BW* body weight, *t-chol* total cholesterol, *TG* triglyceride, *p* phosphorylated, *HE* hematoxylin and eosin.

We assessed body weight (BW), the liver and epididymal fat weight/BW ratio, H&E staining, and liver TG content at the end of the 16-week treatment. Mice fed HF-CDAA gained 2 g and exhibited an increase in liver and epididymal fat/BW ratio (Fig. 1A,B). While serum total cholesterol and TG levels were lower, the AST and ALT values in mice fed a HF-CDAA were substantially higher than those in mice fed standard chow (Fig. 1C). These findings indicated not only that a 45% HF-CDAA diet inhibited very low-density lipoprotein secretion (Supplementary Fig. S2) from the liver and promoted intrahepatic lipid accumulation, but also the addition of HF feeding to CDAA in C57BL/6 J mice was identified as an optimal model of steatohepatitis in the absence of substantial weight loss. Exacerbated liver steatosis was confirmed by H&E staining and by measurement of liver TG content (Fig. 1D,E). Furthermore, to evaluate liver injury and fibrosis, we assessed the lobular inflammatory score, performed a quantitative analysis of the area positive for picro-sirius red, and determined hepatic mRNA levels of type1 α1 procollagen. According to the results, HF-CDAA significantly increased these parameters of liver injury and fibrosis (Fig. 1F–H).

As previously reported, excess intrahepatic lipid accumulation triggered metabolic inflammation and led to impairment of hepatic insulin sensitivity and promotion of ER stress^35,36^. Tumor necrosis factor-α induces phosphorylation of IRS-1 at Ser 636/639 through the both Erk and JNK phosphorylation and inhibits tyrosine phosphorylation of IRS-1^37^. In accordance with these reports, HF-CDAA promoted liver inflammation with phosphorylation of NF-κB p65 at Ser536, that of Erk1/2, and that of JNK (Fig. 1I, Supplementary Fig. S4a–c). At the same time, phosphorylation of AMPKα and of IRS-1 at Tyr896 was reduced, while phosphorylation IRS-1 at Ser636 was elevated (Fig. 1J, Supplementary Fig. S5a–c). The assessment of ER stress-related molecules showed that phosphorylation of eIF2α and CHOP expression were upregulated by HF-CDAA (Fig. 1K, Supplementary Fig. S6a–c). Taken together, the HF-CDAA-induced inflammatory response inhibited hepatic insulin signaling and enhanced the ER stress pathway, which was accompanied by downregulation of AMPK activity.

### GW7647 treatment ameliorated HF-CDAA induced liver steatosis

**Figure 2:**
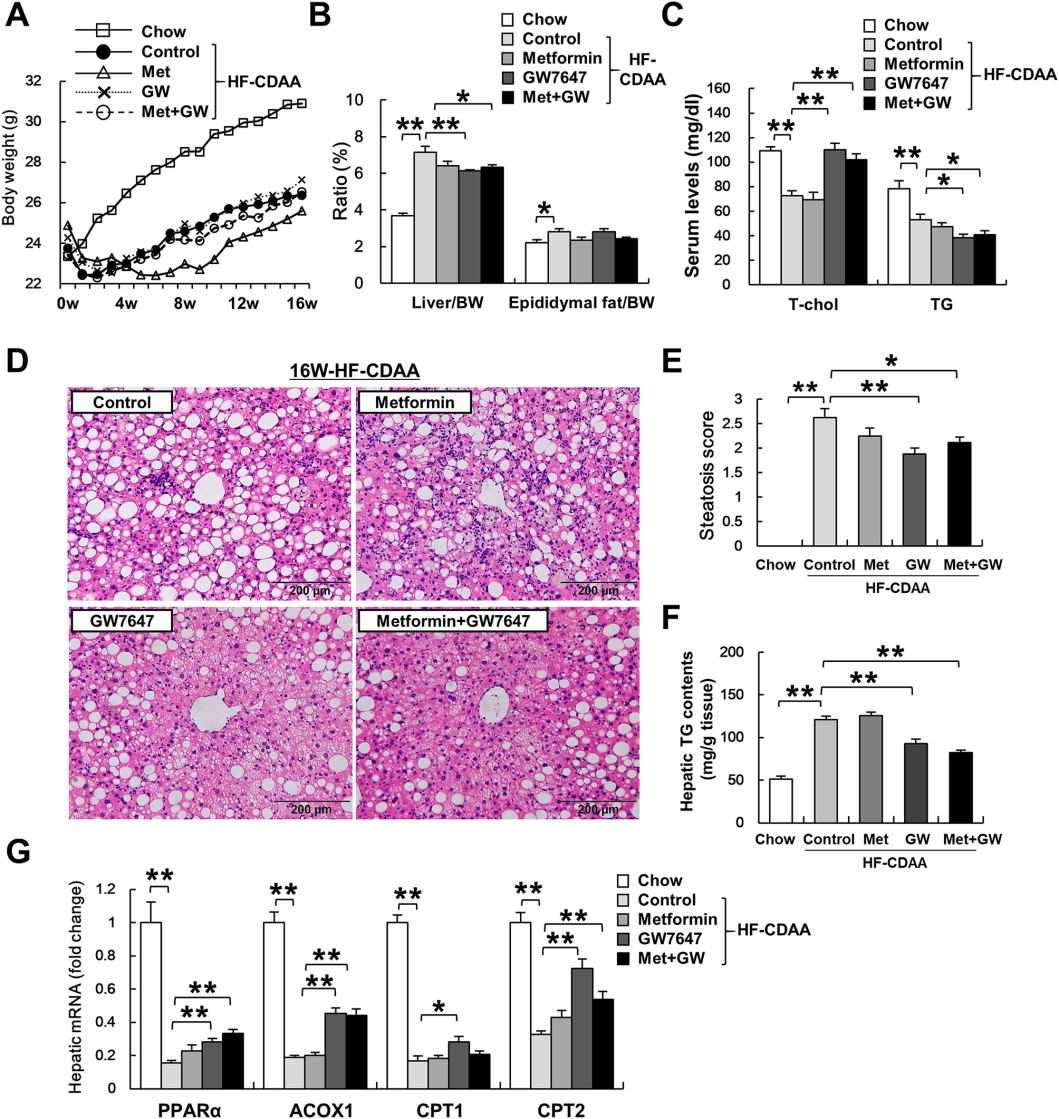
The effect of metformin, GW7647 and metformin/GW7647 on HF-CDAA-induced liver steatosis. (**A**) Body weight changes during 16-week treatment are plotted. (**B**) Liver/BW and epididymal fat/BW ratio (%) were assessed. (**C**) Serum TG were determined in each group (n = 8/group). (**D**) Hematoxylin and eosin staining of liver sections from representative mice fed chow or HF-CDAA diets. (**E**) Liver steatosis grade was scored according to brunt criteria. (**F**) Hepatic TG contents were measured. (**G**) Hepatic mRNA levels of PPARα, ACOX, and CPT1, 2 were determined. Mean ± SE data are displayed as fold changes relative to chow-fed mice. *p < 0.05, **p < 0.01. *Met* metformin, *GW* GW7647.

To investigate the effect of metformin and a PPARα agonist against NASH, we treated mice with metformin, GW7647, or both agents for 16 weeks. The final BWs at the end of the treatment were similar among all groups (Fig. 2A). The liver/BW ratio, but not the epididymal fat/BW ratio, and serum TG levels were significantly decreased by both GW7647 monotherapy and co-treatment with metformin and GW7647 (Fig. 2B,C). Liver steatosis were assessed in H&E stained sections (Fig. 2D). In accordance with a significant decrease in liver TG contents, the liver steatosis grade was clearly improved in the GW7647 monotherapy and co-treatment groups (Fig. 2E,F).

The major mechanism driving hepatic TG accumulation is increased delivery of free fatty acids from peripheral adipose depots to the liver. Hepatic lipid disposal via mitochondrial/ peroxisomal β-oxidation and lipoprotein export are central mechanisms that function to eliminate potentially toxic free fatty acids^35,36,38^. To investigate why liver steatosis was ameliorated by GW7647 monotherapy and co-treatment, we assessed hepatic mitochondrial and peroxisomal β-oxidation-related gene expression of CPT1, 2, and ACOX1 by real-time PCR. Although HF-CDAA diets severely suppressed expression of CPT1, 2 and ACOX1, which are target genes of PPARα, we found that GW7647 treatment partly restored these gene expressions in comparison with HF-CDAA controls (Fig. 2G)^39^. Consistent with previous reports of fibrates, these findings indicated that the GW7647 treatment groups experienced an amelioration in steatosis induced by HF-CDAA via the induction of both hepatic ACOX1, is the first and rate-limiting enzyme in fatty acid β-oxidation in peroxisomal, and CPT1, 2, controls mitochondrial beta-oxidation.

### Co-treatment with GW7647 and metformin ameliorated liver inflammation

**Figure 3:**
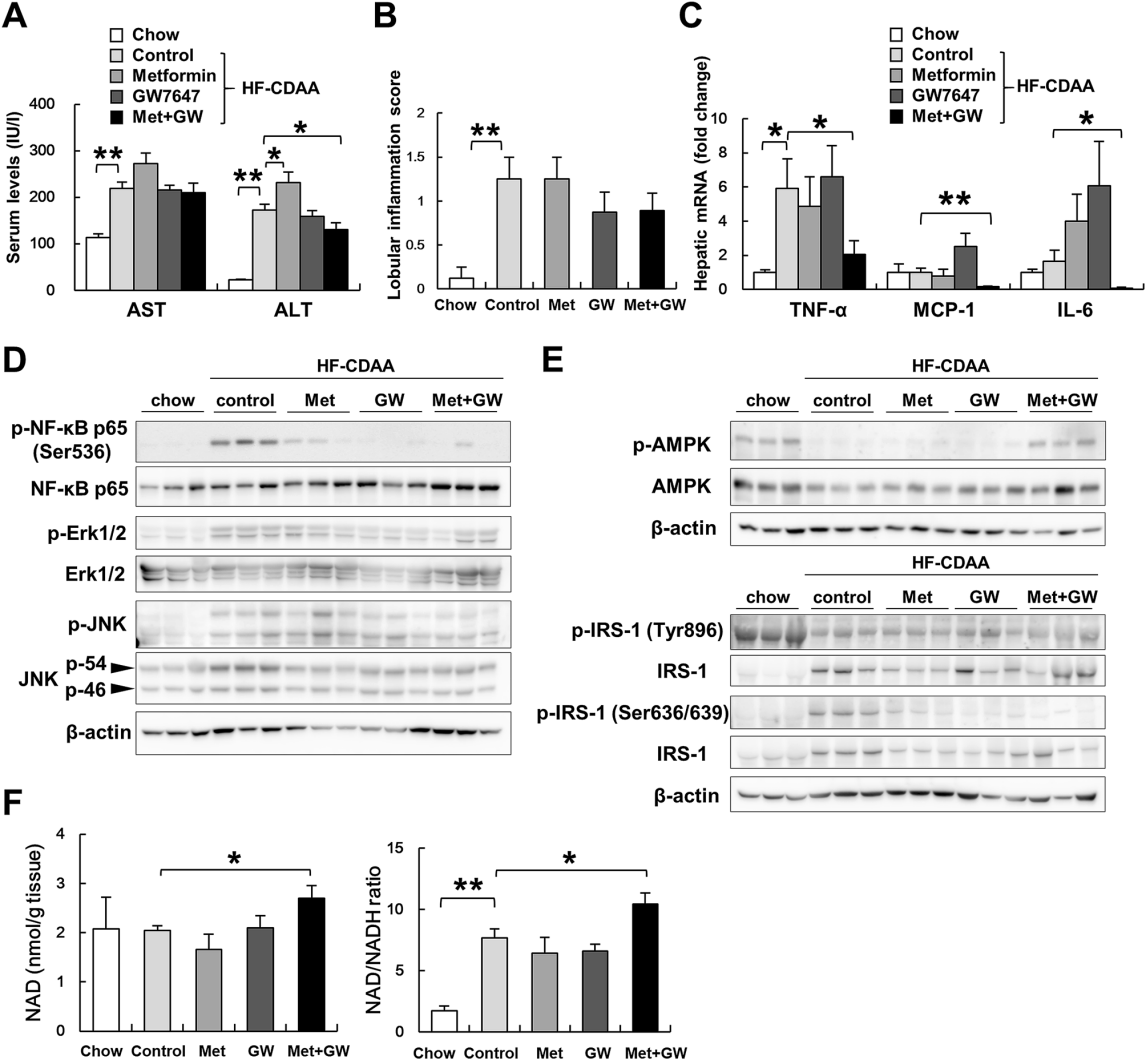
The effect of metformin, GW7647 and metformin/GW7647 on Injury-related parameters. (**A**) Serum AST and ALT values were determined. (**B**) The number of inflammatory foci per 200 × field was counted. (**C**) Hepatic mRNA levels of TNF-α, IL-6 and MCP-1 were determined. (**D**) p-NF-κB p65 at Ser536, t-NF-κB p65, p-Erk1/2 at Thr202/Tyr204, t-Erk1/2, p-JNK at Thr183/Tyr185, t-JNK, (**E**) p-AMPKα at Thr172, t-AMPKα, p-IRS-1 at Tyr896 and at Ser636, and t-IRS-1 levels were evaluated by immunoblot analysis. (**F**) Hepatic NAD and NAD/NADH ratio were measured. Results are expressed per g tissue. *p < 0.05, **p < 0.01. *TNF-α* tumor necrosis factor-alpha, *MCP1* monocyte chemotactic protein 1, *IL-6* Interleukin-6.

Unexpectedly, metformin monotherapy increased the serum AST and ALT values compared with other treatments (Fig. 3A). On the contrary, co-treatment, but not GW7647 monotherapy, ameliorated serum ALT values and tended to lower the lobular inflammatory scores of liver sections (Fig. 3A,B). Hepatic mRNA levels of tumor necrosis factor-alpha, monocyte chemotactic protein-1, and interleukin 6 were also significantly decreased in the co-treatment group, which suggests anti-inflammatory effects of GW7647 and metformin co-treatment (Fig. 3C). To confirm these findings, we used immunoblotting to compare hepatic injury-related parameters. HF-CDAA-induced phosphorylation of NF-κB p65 at Ser536, that of Erk1/2 at Thr202/Tyr204, and that of JNK at Thr183/Tyr185 were abrogated in the livers of mice in the co-treatment group (Fig. 3D). Consistent with these observations, the phosphorylation of AMPKα at Thr172 was restored with a reduction in phosphorylation of IRS-1 at Ser636 by HF-CDAA diets (Fig. 3E), which indicates that the activation of AMPK by metformin inhibited inflammatory stimuli and the phosphorylation of IRS-1 at Ser636 only in the co-treatment group. To investigate why only co-treatment could activate AMPK and ameliorate liver injury, we assessed NAD, which can restore mitochondrial function and energy production, and the NAD/NADH ratio. HF-CDAA diets did not alter the hepatic levels of NAD but increased NAD/NADH ratio because of the decreases of NADH levels. Both NAD and the NAD/NADH ratio were elevated in the co-treatment group but not in the other HF-CDAA groups (Fig. 3F).

### Co-treatment with GW7647 and metformin ameliorated ER stress

**Figure 4:**
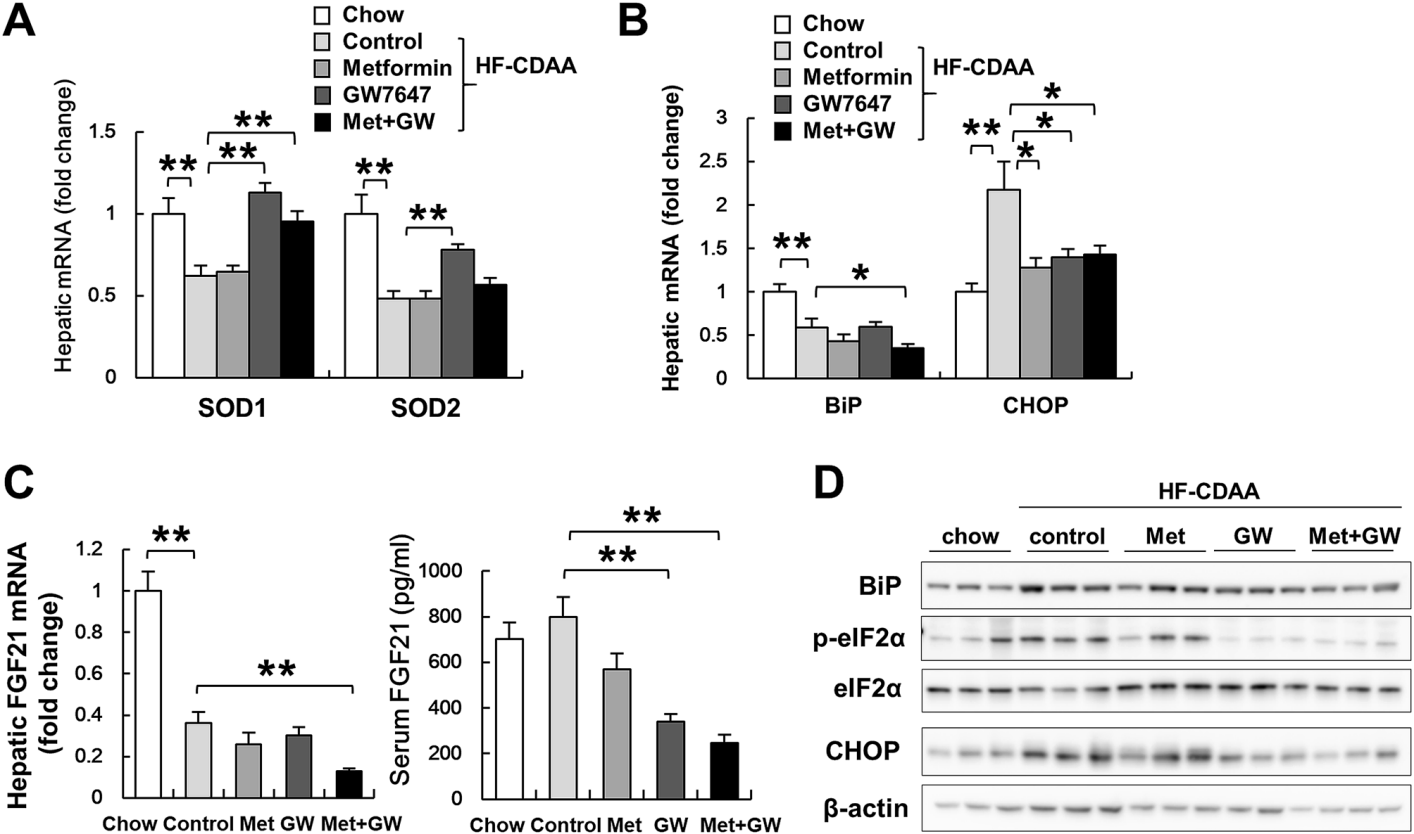
The effect of metformin, GW7647 and metformin/GW7647 on ER-stress-related parameters. (**A**) Hepatic mRNA levels of SOD1, 2 (**B**) BiP, and CHOP were determined by quantitative real time PCR analysis. (**C**) Hepatic mRNA levels and serum levels of FGF21 were determined in each group (n = 8/group). (**D**) BiP, p-eIF2α at Ser51, t-eIF2α and CHOP levels were evaluated by immunoblot analysis. *p < 0.05, **p < 0.01. *SOD* superoxide dismutase, *BiP* immunoglobulin heavy chain-binding protein.

We also assessed oxidative/ER stress parameters. While HF-CDAA diets suppressed the mRNA levels of superoxide dismutase-1 and 2, which are target genes of PPARα, GW7647 treatment clearly restored these levels (Fig. 4A). ER stress molecules and immunoglobulin heavy chain-binding protein mRNA were lower in HF-CDAA groups than those in chow-fed mice, and lowest in the co-treatment group (Fig. 4B). On the contrary, CHOP mRNA was elevated by HF-CDAA diets and suppressed in all three treatment groups (Fig. 4B).

These observations indicated that co-treatment might be an effective treatment for the induction of PPRE-dependent anti-oxidant gene expression and for improving expression of ER stress-related molecules. While hepatic mRNA levels of FGF21 were lower in HF-CDAA groups compared to chow group, interestingly, hepatic mRNA levels of FGF21 and serum FGF21 levels were significantly decreased in mice treated with both agents (Fig. 4C). These results were inconsistent with previous studies that showed that the FGF21 gene is a PPARα target^40^. Therefore, we evaluated the protein levels of immunoglobulin heavy chain-binding protein, phosphorylated eIF2α, and CHOP, which also regulate FGF21 expression. Despite of no change in immunoglobulin heavy chain-binding protein levels between all groups, we found that co-treatment actually reduced the elevation of phosphorylated eIF2α and CHOP by HF-CDAA diets (Fig. 4D), suggesting that the improvement of ER stress led to a reduction of FGF21 expression.

### Co-treatment with GW7647 and metformin ameliorated liver fibrosis

**Figure 5:**
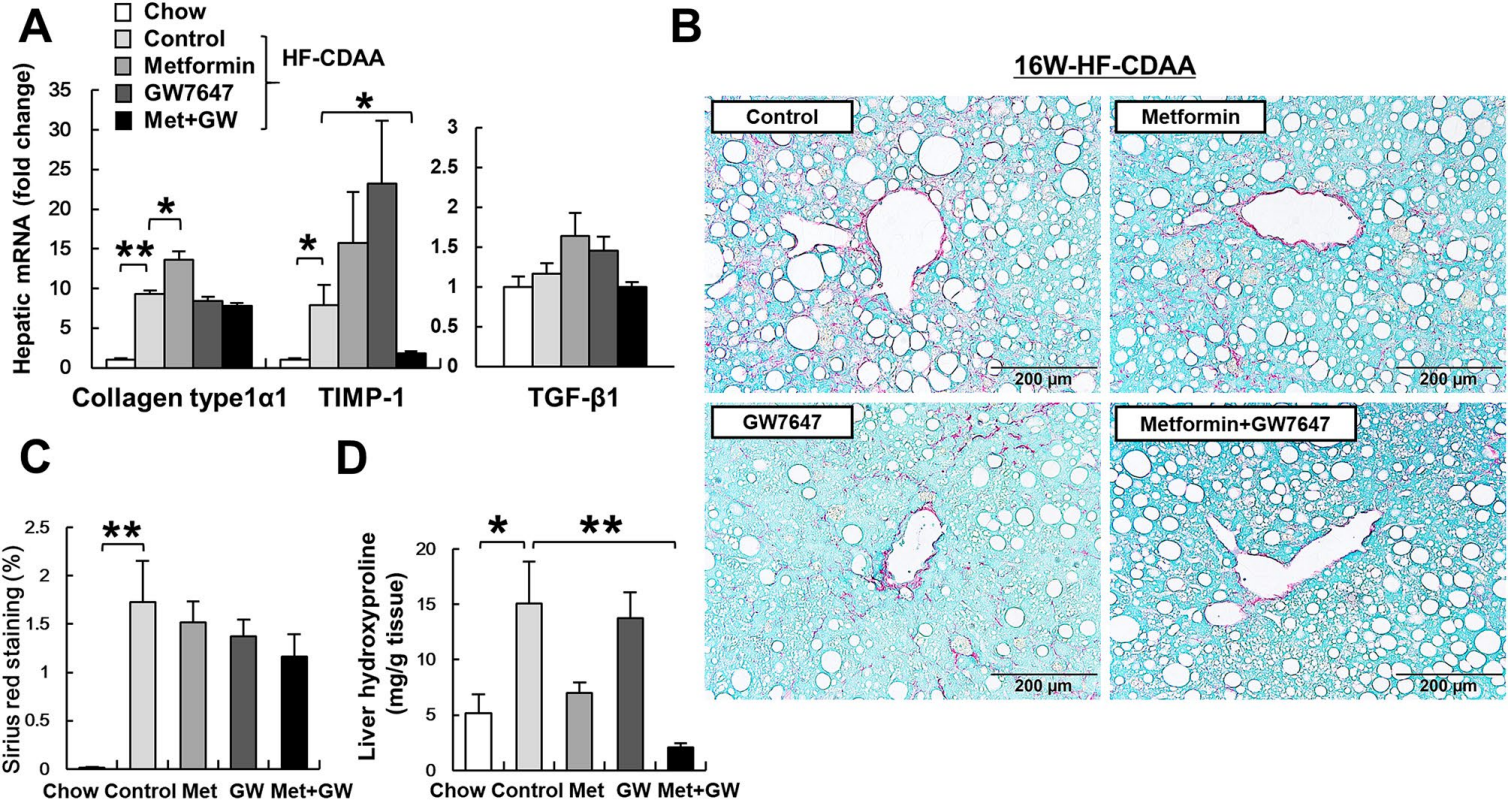
The effect of metformin, GW7647 and metformin/GW7647 on fibrosis markers. (**A**) The mRNA levels of hepatic collagen, TIMP-1 and TGF-β1 were determined by quantitative real time PCR. (**B**) Picro-sirius red staining of liver sections from representative mice of each group. (**C**) Morphometric analysis of picro-sirius red-stained sections from each group (n = 8/group). Results are expressed as percentage of section staining (+) for picro-sirius red. (**D**) Hepatic hydroxyproline contents were measured. Results are expressed per g tissue. *p < 0.05, **p < 0.01. *TIMP-1* tissue inhibitor of metalloproteinase-1, *TGF-β1* transforming growth factor-beta 1.

To evaluate the effect of co-treatment on liver fibrosis, hepatic mRNA levels of fibrosis markers were compared among the four groups. Mice that were fed the HF-CDAA diet exhibited significantly increased hepatic mRNA expression of collagen type 1 α 1, tissue inhibitor of metalloproteinase-1, and transforming growth factor-beta 1. Co-treatment suppressed these HF-CDAA diet-related increases in fibrosis makers (Fig. 5A). To further assess the effects of co-treatment on hepatic fibrosis, picro-sirius red staining and a measurement of liver hydroxyproline content were performed (Fig. 5B–D). As predicted from the results of the analysis of hepatic fibrosis markers, co-treatment slightly decreased the proportion of picro-sirius red-stained areas, as demonstrated by liver morphometry, and significantly lowered liver hydroxyproline content in mice. These findings demonstrated the anti-fibrotic effect of co-treatment in this model of advanced NASH.

## Discussion

Both metformin and PPARα agonists are well tolerated and commonly used in patients with obesity-related metabolic disorders, such as type 2 diabetes mellitus and hyperlipidemia. Several studies have already shown that they can also induce additional benefits for NAFLD^11–13,15,17^. However, whether they improve the histology of NAFLD has not been fully elucidated. In this study, we observed the beneficial effect of co-treatment with a PPARα agonist and metformin on the progression of NAFLD in three major manifestations of liver disease, that is, steatosis, inflammation, and fibrosis. We concluded that co-treatment actually helps to protect hepatocytes from metabolic inflammation by buffering impaired mitochondrial function in this mouse model of advanced NASH.

Like methionine-choline-deficient diets, CDAA diets induce hepatic TG accumulation by inhibiting hepatic export of very low-density lipoproteins and impairing fatty acid oxidation in hepatocytes^29^. These inhibitory effects on lipid disposal are sufficient to increase lipid synthesis as well as oxidative and endoplasmic reticulum stress to incite hepatic inflammatory cell infiltration and stellate cell activation^29^. Interestingly, unlike high-fat diets, which produce relatively little hepatic necroinflammation or fibrosis, CDAA diets are widely used to induce NASH with fibrosis, even in normal mice. However, although CDAA diets have been reported to induce peripheral insulin resistance at one month^41^, lean mice fed CDAA diets for long periods display neither obvious changes in peripheral insulin sensitivity nor weight gain^29^. Therefore, we used a CDAA diets containing 45% fat, by which mice gain 2 g for 16 weeks and exhibit impaired hepatic insulin signaling via phosphorylation of IRS-1 at Ser636/639. HF-CDAA-fed mice demonstrate steatohepatitis with dietary fat-driven dysregulation of lipid metabolism-related genes, progressive fibrosis, and hepatocellular carcinoma^29^. In our study, mice fed an HF-CDAA diet for 16 weeks gained an average of 2 g of weight without concomitant increases in serum glucose and insulin levels (Supplementary Fig. S3). In their livers, while phosphorylation of IRS-1 at Tyr896 and total IRS-1 was decreased, phosphorylation of IRS-1 at Ser636 was significantly increased, which suggests that this HF-CDAA diet-induced metabolic inflammation that was involved in the impairment of hepatic insulin signaling (Fig. 1J)^37,42–44^. Here, we tested the anti-diabetic drug metformin, which has potential tumor suppressive effects. Unfortunately, metformin monotherapy failed to enhance the phosphorylation of AMPKα at Thr172 and worsened serum AST/ALT values and hepatic collagen mRNA (Fig. 3A, E, Fig. 5A). In contrast, GW7647 monotherapy expectedly upregulated hepatic expression of PPRE-dependent and PPRE-independent genes and tended to ameliorate liver steatosis, but these effects were not sufficient to prevent liver fibrosis. Co-treatment with GW7647 and metformin successfully improved NASH through beneficial effects on the metformin-AMPK axis and PPARα activity in this model of advanced NASH.

AMPK is a serine/threonine protein kinase. AMPK signaling is a cellular energy and nutrient sensor, and plays an essential role in metabolic pathways. Metformin activates the AMPK pathway and inhibits phosphorylation of mechanistic target of rapamycin^18–21^. We considered that the metformin-induced anti-inflammatory effect was partly accompanied by upregulation of AMPKα phosphorylation (Thr172), downregulation of Erk1/2 (Thr202/Tyr204), and JNK (Thr183/Tyr185) and IRS-1 (Ser636) phosphorylation in the co-treatment group. Consistent with these results, it is known that the inhibitory effect of metformin on mitogen-activated protein kinase activity is involved in protection against cardiovascular diseases, atherosclerosis, and chronic kidney disease. Recently, imeglimin, is a new anti-diabetes drug, inhibits complex I, and restores complex III activities, which leads to an increase in fatty acid oxidation and a reduction in liver steatosis. This dual effect of imeglimin (complex I inhibition and complex III restoration) allows mitochondria to oxidize more complex II substrates, and thus, potentially more lipids^45^. We observed that co-treatment, but not metformin monotherapy, elevated the production of NAD and the NAD/NADH ratio in the liver, which indicates benefits of co-treatment on impaired mitochondrial function. In summary, in this study, metformin could restore mitochondrial function and inhibit liver inflammation by the addition of a PPARα agonist. Metformin was also reported to inhibit the invasiveness of human hepatocellular carcinoma cells via downregulation of Erk/JNK-mediated NF-κB-dependent signaling^18,22,23^. Therefore, hepatocellular carcinoma induced by long-term of HF-CDAA feeding might be suppressed by this co-treatment.

PPARα activation also inhibits inflammatory genes induced by NF-κB and decreases the expression of acute-phase response genes in a PPRE-dependent or PPRE-independent manner^12^. Fibrates, which are commonly used as PPAR agonists, maintained the elevated de novo lipogenesis (indicated by the upregulation of sterol regulatory element-binding protein 1c, acetyl-CoA carboxylase, fatty acid synthase, and stearoyl-CoA desaturase 1), markedly increased fatty acid oxidation (indicated by induction of ACOX1, phospho-acetyl-CoA carboxylase) activity, and eliminated intrahepatic lipid accumulation^12,46^. Consistently, GW7647 increased de novo lipogenesis-associated genes and serum total cholesterol levels (Fig. 2C, Supplementary Fig. S3c), and enhanced the expression of fatty acid oxidation-related genes (Fig. 2G) and cyp7a1 (Supplementary Fig. S3c). Finally, activation of PPARα can reduce the accumulation of deleterious lipids and lead to amelioration of hepatic insulin resistance and oxidant/ER stress^13,47^. Therefore, due to the pathophysiological role of PPARs in NAFLD, these data suggest that PPARα is a potential therapeutic target in NASH. In clinical studies, the use of PPARγ agonists is associated with histological improvement in NAFLD but is harmful for obesity^48^. In other studies, human liver PPARα gene expression was negatively correlated with NASH severity, visceral adiposity, and insulin resistance and was positively correlated with adiponectin^49^. Histological improvement is associated with an increase in the expression of PPARα and its target genes. Upregulation of PPARα and its target genes in patients with NASH at baseline was correlated with a significant downregulation of inflammatory response genes, as well as that of lipogenesis genes in responders to fibrate treatment^49^. Moreover, FGF21 is strongly induced in animal and human subjects with metabolic diseases, is expressed predominantly in the liver, and has recently emerged as a promising drug candidate for NASH^50,51^. In our study, hepatic FGF21 expression and serum FGF21 levels were lowest in the co-treatment group (Fig. 4D). At a glance, our results differ from those of some reports that have stated that FGF21 is a key downstream mediator of PPARα-induced effects^40^. However, FGF21 is also known to be regulated by ER-stress-related genes^51^, such are activating transcription factor 4 and CHOP, which suggests that the amelioration of ER stress by co-treatment reduced FGF21 expression in the liver.

Metformin monotreatment suppressed phosphorylation of NF-κB p65, Erk1/2 and eIF2α, but not led to an amelioration of NASH. GW7647 monotreatment also suppressed phosphorylation of NF-κB p65, IRS-1 (at ser 636/639) and eIF2α, but not clearly ameliorated NASH. The possibility remains that higher dose of metformin or GW7647 was more effective. Because average food intakes were almost 3–4 g/day in all groups (Supplementary Fig. S3a), we had selected the minimum quantity of an effective dose in both metformin and GW7647. Metformin (0.1% w/w in standard diet) could activate AMPK without altering in vivo electron transport chain activities^31^. And 2.5 mg/kg/d of GW7647 is sufficient to induce its effects as a PPARα agonist according to Li et al.^32^. We were fearful of high dose of PPARα agonist-related hepato-carcinogenesis in the HF-CDAA models. In this study, our aim is how to use metformin safely and effectively for the treatment of advanced NASH. The minimum and effective quantity dose of PPARα agonists and metformin is a probable candidate for NASH therapy. Upon consideration of the results of the present study and those of other reports, we conclude that co-treatment can prevent the progression of NASH. This is due to an additive interaction of the beneficial effects of co-treatment on metabolic inflammation and mitochondrial function. Recently, dual PPAR agonists without significant gamma activity have appeared promising for the treatment of NAFLD^52^. Hopefully, further studies on the therapeutic use of PPAR agonists and metformin, including their related drugs, for the treatment of NASH will be performed in the future.

## Methods

### Animals and treatment

A mouse model of diet-induced obesity and NASH was used in this study. Eight-week-old male C57/BL6 mice were purchased from Japan Jackson Laboratories, maintained in a temperature- and light-controlled facility, permitted consumption of water ad libitum, and housed in transparent polymer X (TPX) cages (CL-0104-2, CLEA Japan Inc., Tokyo, Japan) with a maximum of eight mice per cage. Eight mice were fed a control chow diet and 32 mice were fed a high-fat CDAA diet (HF-CDAA; A06071309, Research Diet Inc., Tokyo, Japan) for 16 weeks. 32 mice fed HF-CDAA diets were randomly divided into four groups (n = 8/group): no treatment control (HF-CDAA) group, the group fed HF-CDAA containing 1000 mg/kg metformin (Sigma, Japan)^31^, the group fed HF-CDAA containing 10 mg/kg GW7647 (Sigma Japan)^32^, and the group fed HF-CDAA containing both metformin and GW7647. Optimized doses of GW7647 were determined by measuring liver concentrations of GW7647 one week after feeding (Supplementary Fig. S1a). Furthermore, to confirm the induction of PPRE-dependent transcriptional activity, hepatic mRNA levels of PPARα, carnitine palmitoyltransferase (CPT)1, and acyl-CoA oxidase (ACOX)1 were examined one week after they were fed a HF-CDAA containing 1 mg/kg, 3 mg/kg, or 10 mg/kg of GW7647. Finally, we decided 10 mg/kg dose of GW7647 is most effective for a biological response. (Supplementary Fig. S1b). At the end of treatment, livers, epididymal fats, and blood were isolated from each animal after about 8 h of fasting. Co-treatment with metformin and GW7647 had no effect on serum concentration of those each other (Supplementary Fig. S1c). The study protocol was in accordance with the guidelines for the care and use of laboratory animals set by Kyoto Prefectural University of Medicine. (Kyoto, Japan) and was approved by the Committee on the Ethics of Animal Experiments of the same institution. Animals were housed under conventional conditions with controlled temperature, humidity, and light (12-h light–dark cycle) and provided with food and water.

### Analysis of liver architecture

Liver sections were stained with hematoxylin and eosin using standard techniques. The steatosis score was calculated according to the degree of parenchymal involvement as follows: 0, < 5%; 1, > 5–33%; 2, > 33–66%; and 3, > 66% according to Brunt criteria^33^. The lobular inflammation score was calculated according to the numbers of the inflammation foci in a × 200 microscopic field as follows: 0, no foci; 1, < 2 foci per × 200 field; 2, 2–4 foci per × 200 field; and 3, > 4 foci per × 200 field.

### Quantification of collagen levels in the liver

To quantify the collagen content in the liver, liver sections were stained with picro-sirius red and counterstained with fast green (Sigma-Aldrich, Japan). The proportion of tissue stained with picro-sirius red was quantified by morphometric analysis using Image J software in five randomly selected fields per section (magnification × 200), as previously described^34^. Hydroxyproline content in whole-liver specimens was quantified using a Total Collagen Assay Kit (QuickZyme BioSciences B.V, Netherlands).

### Two-step real-time PCR

Real-time PCR was performed as described below^34^. Specificity was confirmed for all primer pairs (Supplementary Table S1) by sequencing the PCR products. Target gene levels were presented as a ratio of levels in the treated versus corresponding control groups. Fold changes were determined using point and interval estimates.

### Immunoblot assay

Proteins isolated from whole livers were separated by SDS–PAGE and transferred to PVDF membranes. Membranes were probed with anti-eukaryotic initiation factor 2α (eIF2α), antiphospho-eIF2α, anti-C/EBP-homologous protein (CHOP), anti-immunoglobulin heavy chain-binding protein, anti-AMPK, anti-phospho-AMPK at Thr172, anti-insulin receptor substrate 1 (IRS-1), anti-phospho-IRS-1 at Ser636, anti-phospho-NF-κB p65 at Ser 536, anti-NF-κB p65, anti-phospho- extracellular signal-regulated kinase (Erk)1/2, anti-Erk1/2, anti-phospho-JNK, anti-JNK (Cell Signaling Technology Inc., Beverly, MA), anti-phospho-IRS-1 at Tyr896 (Sigma-Aldrich), or anti-actin (Sigma-Aldrich), followed by incubation with horseradish peroxidase-conjugated anti-mouse or anti-rabbit IgG (Invitrogen, Carlsbad, CA). Antigens were visualized by ECL (GE Healthcare, Chicago, IL).

### Tissue and plasma biochemical measurements

Serum AST, ALT, total cholesterol, and TG levels were measured at SRL, Inc. (Tokyo, Japan). Serum FGF21 levels were measured using a Mouse FGF21 ELISA Kit (Arigo Biolaboratories Corp., Hsinchu, Taiwan). We performed the extraction of hepatic lipids from about 20 mg liver tissue of each sample by Bligh & Dyer methods, and remeasured liver TG contents using the commercial kits, triglyceride E-test (Wako Pure Chemical Industries). The hepatic NAD/NADH ratio was measured by NAD/NADH Quantification Colorimetric Kit (BioVision, Inc., CA).

### Statistical analysis

Results are presented as the mean ± SEM. Significance was established using Student’s t-test and analysis of variance, when appropriate. Differences were considered significant if p < 0.05.

## Supplementary information

Supplementary Information

## Abbreviations

ACOX: Acyl-CoA oxidase
ALT: Alanine aminotransferase
AMPK: Adenosine monophosphate-activated protein kinase
AST: Aspartate aminotransferase
CDAA: Choline-deficient l-amino acid-defined
CHOP: C/EBP-homologous protein
CPT: Carnitine palmitoyltransferase
eIF2α: Eukaryotic initiation factor 2-alpha
ER: Endoplasmic reticulum
Erk: Extracellular signal-regulated kinase
FGF21: Fibroblast growth factor 21
HF: High-fat
IRS-1: Insulin receptor substrate 1
JNK: C-jun N-terminal kinase
NAD: Oxidized nicotinamide adenine dinucleotide
NADH: Reduced nicotinamide adenine dinucleotide
NAFLD: Nonalcoholic fatty liver disease
NASH: Nonalcoholic steatohepatitis
NF-κB: Nuclear factor-kappa B
PPAR: Peroxisome proliferator-activated receptor
PPRE: Peroxisome proliferator response element
TG: Triglyceride
