# Supplementary material for: PPARα agonist and metformin co-treatment ameliorates NASH in mice induced by a choline-deficient, amino acid-defined diet with 45% fat
Source: Sci Rep. 2020 Nov 11;10:19578. doi: 10.1038/s41598-020-75805-z (PMC7658250; doi:10.1038/s41598-020-75805-z)
Supplement: Supplementary file 1 — Supplementary Information [file 41598_2020_75805_MOESM1_ESM.pdf]

SUPPLEMENTARY INFORMATION

PPARα agonist and metformin co-treatment ameliorates NASH in mice induced by a choline-deficient, amino acid-defined diet with 45% fat

Shinya Okishio, Kanji Yamaguchi, Hiroshi Ishiba, Nozomi Tochiki, Kota Yano、Aya Takahashi, Seita Kataoka, Keiichiroh Okuda,Yuya Seko, Yu Liu, Hideki Fujii, Daiki Takahashi, Yusuke Ito, Junji Kamon, Atsushi Umemura, Michihisa Moriguchi, Kohichiroh Yasui, Takeshi Okanoue, and Yoshito Itoh

Contents:

1. Supplementary Table 1. Real time PCR primers for analysis.
2. Supplementary Figures and Legends.

1. Supplementary Table 1. Real time PCR primers for analysis.

	Direction	Sequence
ACOX1	Forward	GCCCAACTGTGACTTCCATC
	Reverse	GCCAGGACTATCGCATGATT
BiP	Forward	ACATGGACCTGTTCCGCTCTA
	Reverse	TGGCTCCTTGCCATTGAAGA
Gene	Forward	CCACCACACCTGAAAGCAGAA
	Reverse	GGTGCCCCCAATTTCATCT
Collagen type 1α1	Forward	GACATCCCTGAAGTCAGCTGC
	Reverse	TCCCTTGGGTCCCTCGAC
CPT1	Forward	TCCATGCATACCAAAGTGGA
	Reverse	TGGTAGGAGAGCAGCACCTT
CPT2	Forward	GCCCAGCTTCCATCTTTACT
	Reverse	CAGGATGTTGTGGTTTATCCGC
CYP7A1	Forward	GGGAATGCCATTTACTTGGA
	Reverse	GTCCGGATATTCAAGGATGC
FAS	Forward	TACCAGTGCCACAGGAGTCTCA
	Reverse	CGGGTGAGGACGTTTACAAAG
FGF21	Forward	CTGGGGGTCTACCAAGCATA
	Reverse	CACCCAGGATTTGAATGACC
GUS	Forward	GCAGTTGTGTGGGTGAATGG
	Reverse	GGGTCAGTGTGTTGTTGATGG
IL-6	Forward	CCGGAGAGGAGACTTCACAG
	Reverse	TCCACGATTTCCCAGAGAAC
MCP-1	Forward	TCAGCCAGATGCAGTTAACGC
	Reverse	TCTGGACCCATTCCTTCTTGG
MTP1	Forward	TGAGCGGCTATACAAGCTCAC
	Reverse	CTGGAAGATGCTCTTCTCGC
PPARα	Forward	GCAGGTCGTACAGGTCATCA
	Reverse	ACTGCCGTTGTCTGTCACTG
SOD1	Forward	CGGCTTCTCGTCTTGCTCTC
	Reverse	CGAAGTGGATGGTTCCCTGC
SOD2	Forward	CAGACCTGCCTTACGACTATGG
	Reverse	CTCGGTGGCGTTGAGATTGTT
Srebp-1c	Forward	GCGGCTACCGGTCTTCTATCA
	Reverse	GGATGTAGTCGATGGCCTTG
TGF-β1	Forward	TTGCCCTCTACAACCAACACAA
	Reverse	GGCTTGCGACCCACGTAGTA
TIMP-1	Forward	CATGGAAAGCCTCTGTGGATATG
	Reverse	GATTGTGCAAATTTCCGTTCCTT
TNF-α	Forward	AAGCCTGTAGCCCACGTCGTA
	Reverse	AGGTACAACCCATCGGCTGG

2. Supplementary Figures and Legends

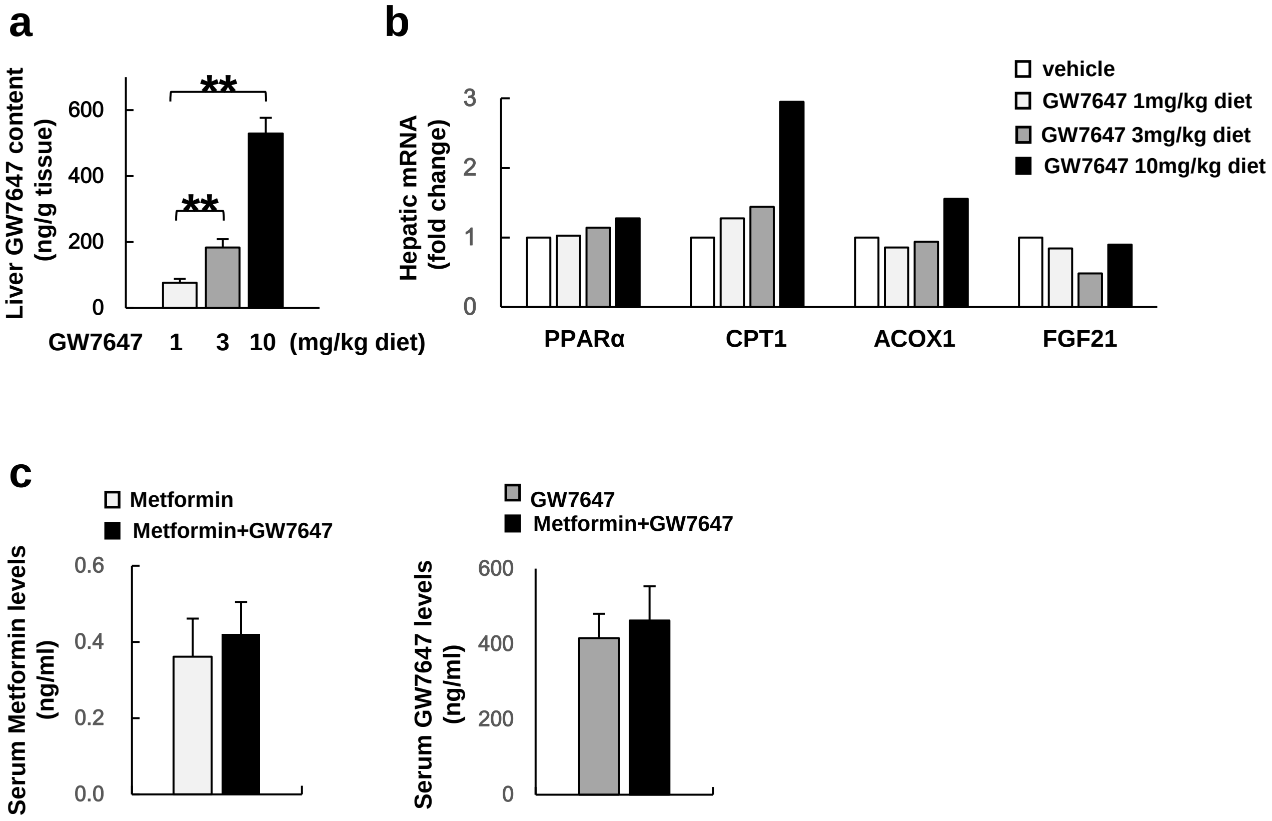

Supplementary Figure S1. Optimized doses of GW7647 in HF-CDAA.
(a) Liver GW7647 contents after one week-feeding of HF-CDAA diets containing 1mg/kg, 3mg/kg and 10mg/kg GW7647 were measured. Results are expressed per g tissue. Data are presented as Mean ± SE from each group (n=3/group). (b) Hepatic mRNA levels of PPARα, CPT1 and ACOX1 were examined. Results were normalized to glucuronidase expression from each group (n=2/group) (c) At the end of treatments, serum concentration of metformin and GW7647 were measured (n=8/group). **p<0.01.

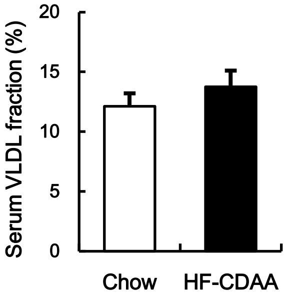

Supplementary Figure S2. Serum VLDL were measured in chow and control (Hf-CDAA) groups.

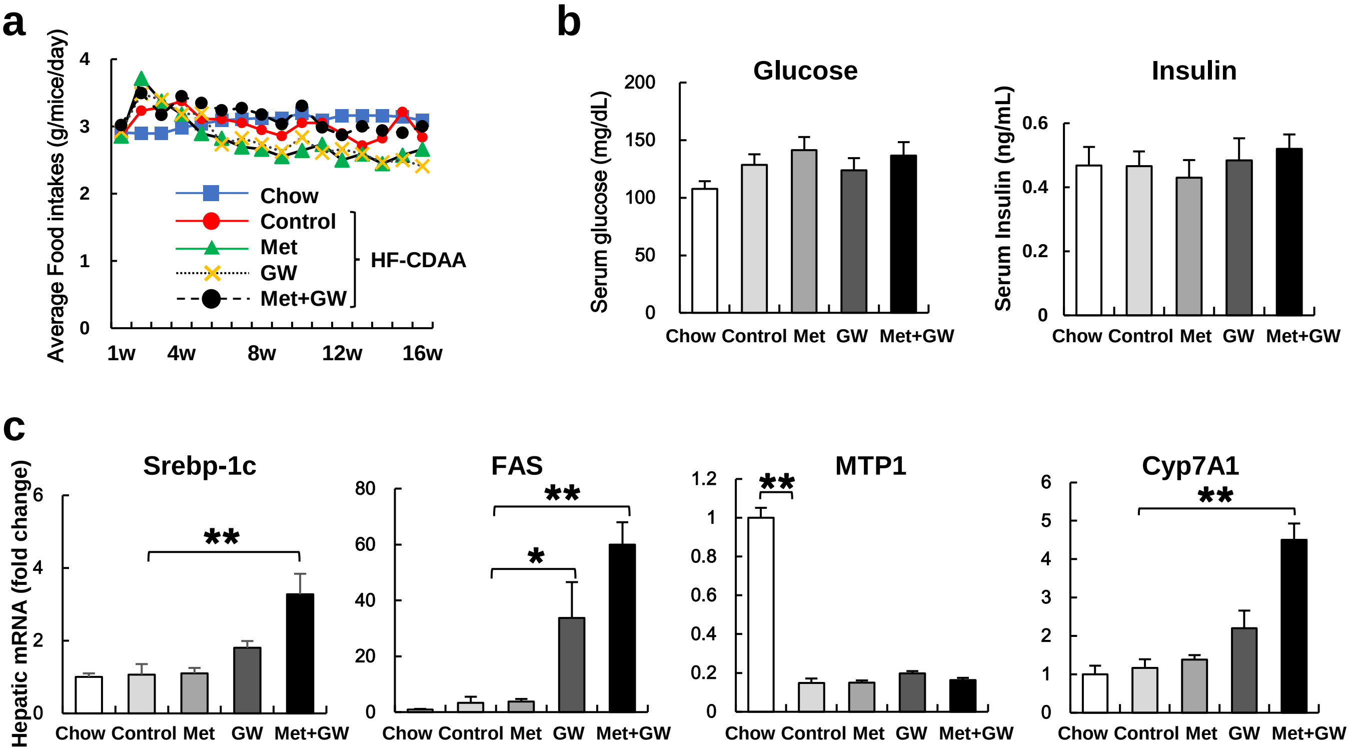

Supplementary Figure S3.
(a) Average food intakes in each group are graphed. (b) Serum glucose and insulin were measured in each group (n=8/group). (c) Hepatic mRNA levels of Srebp-1c, FAS and MTP1 and cyp7A1 were examined. Results were normalized to glucuronidase expression from each group (n=8/group). *p<0.05, **p<0.01.

a **p-NF-κB p65 (Ser536) and NF-κB p65 of Figure 3D**

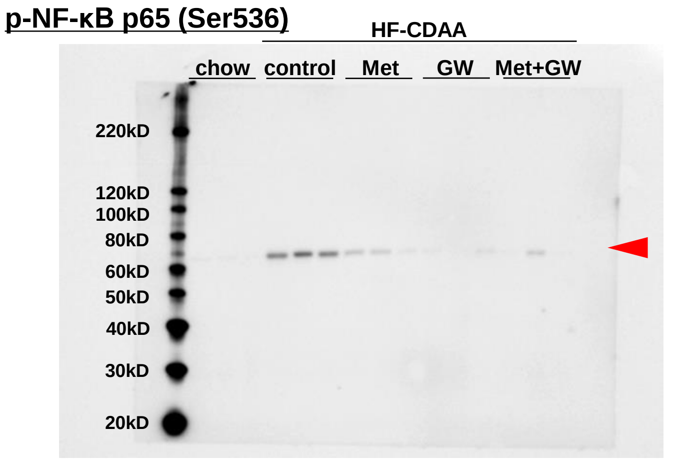

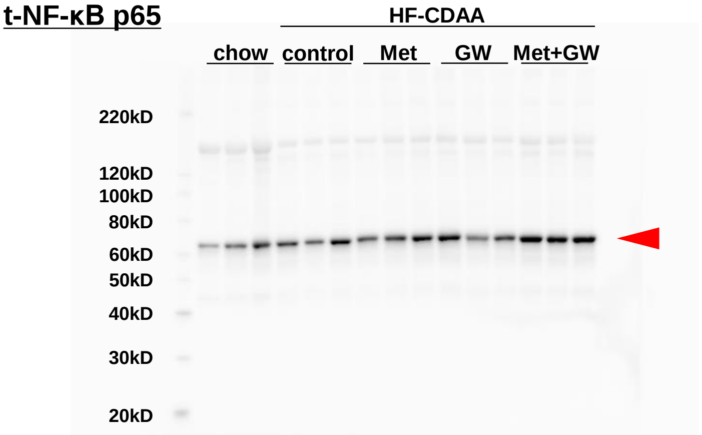

The blot was stripped and re-probed for NF-κB p65 (↑) and β-actin (↓).

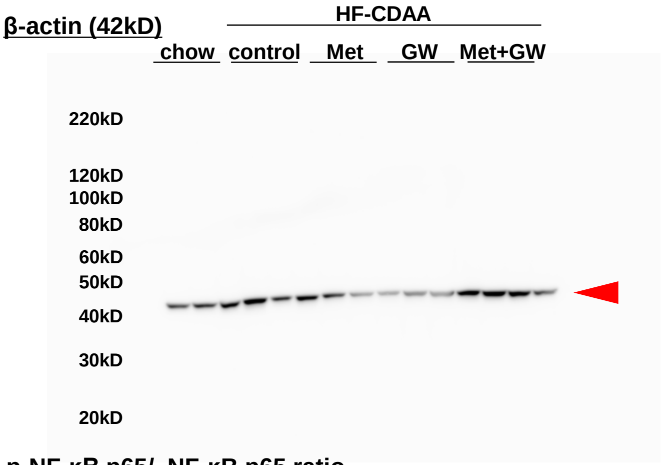

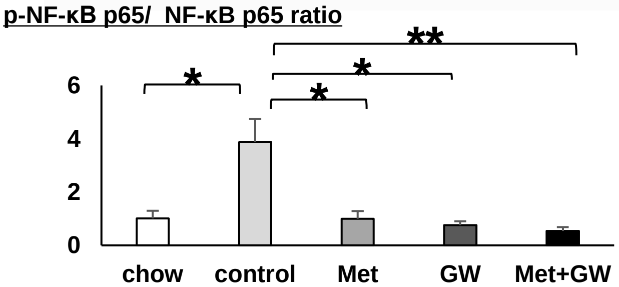

b **p-Erk1/2 and Erk of Figure 3D**

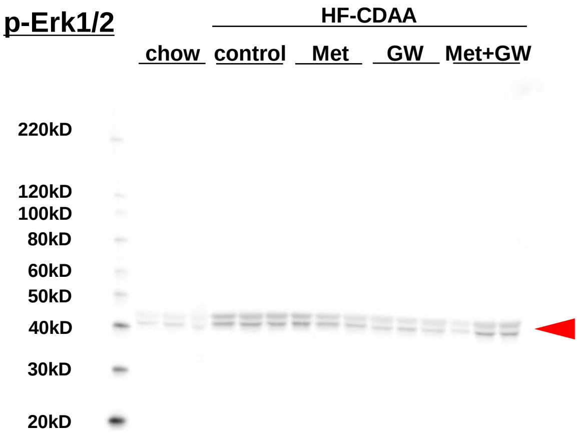

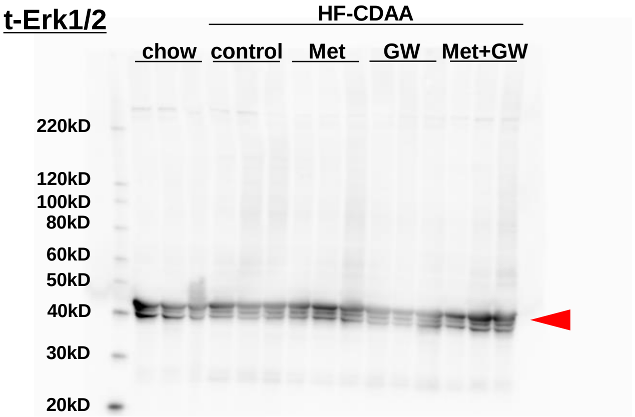

The blot was stripped and re-probed for Erk1/2 (↑) and β-actin (↓).

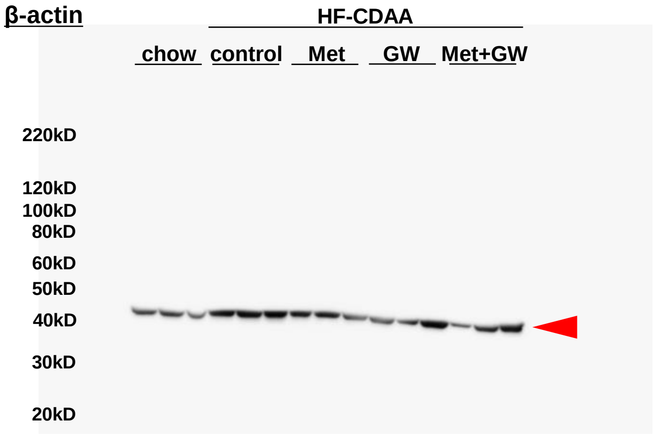

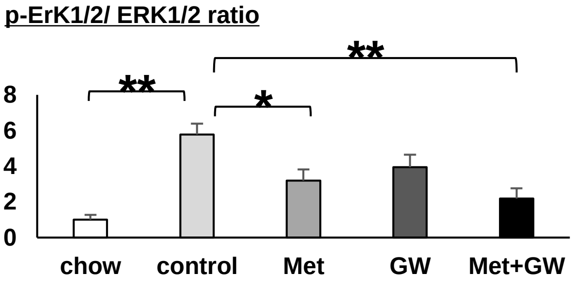

Phosphorylated(p)- NF-κB/NF-κB and p-Erk/Erk ratios in each group were expressed as fold change of chow (*p<0.05, **p<0.01).

c **p-JNK, JNK and β-actin of Figure 3D**

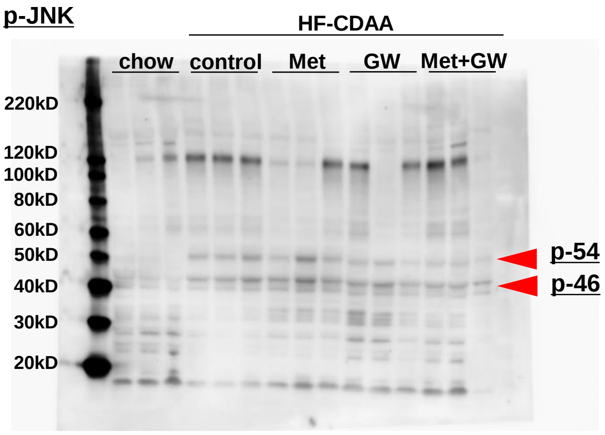

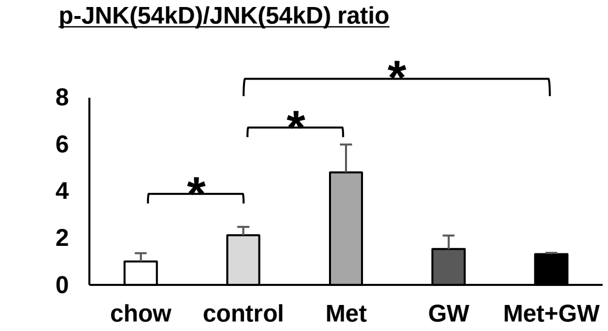

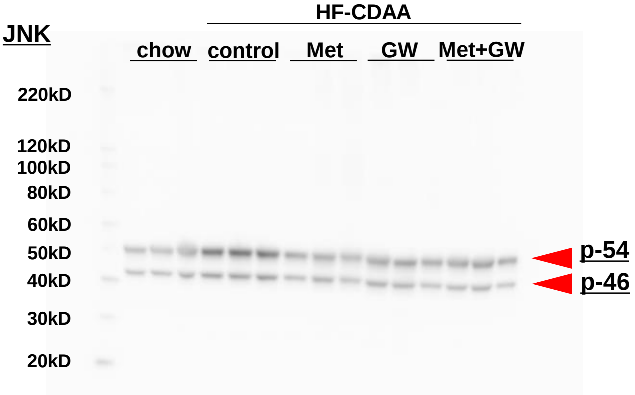

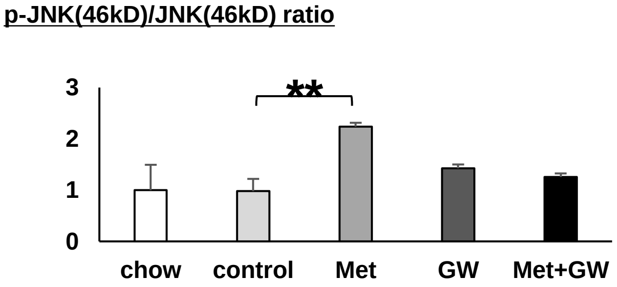

P-JNK/JNK(p54 and p46) ratios were expressed as fold change of chow (*p<0.05, **p<0.01).

The blot was stripped and re-probed for JNK (↑)) and β-actin (↓).
.

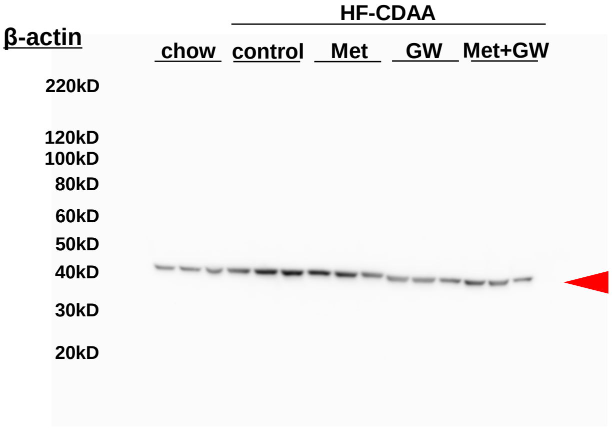

Supplementary Figure S4. Uncropped images for which boxed areas are shown in the published panels I of Figure 1and panels D of Figure 3. (a) Phosphorylated(p)-NF-κB p65/ NF-κB p65, (b) p-ErK1/2/ ERK1/2 and (c) p-JNK(46, 54kD)/JNK(46, 54kD) levels were evaluated by immunoblot analysis of livers from 3 mice/group. Immunoblots of p-NF-κB p65/ NF-κB p65, p-ErK1/2/ ERK1/2 and p-JNK(46, 54kD)/JNK(46, 54kD) were scanned, and band intensities were quantified by Image J (NIH) densitometry analysis. Each ratios were also expressed as fold change of controls. *p<0.05, **p<0.01.

a p-AMPK, AMPK and β-actin of Figure 3E

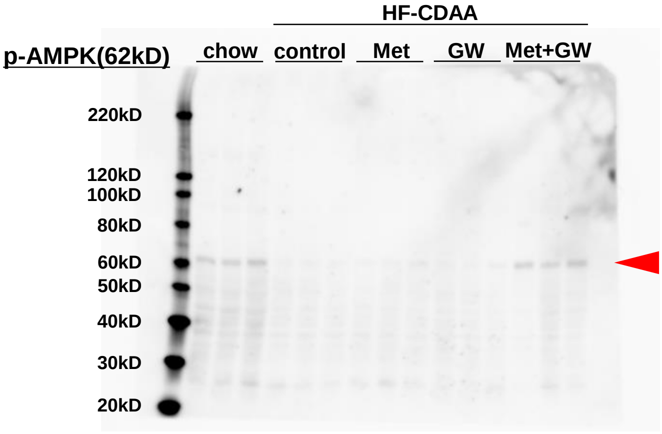

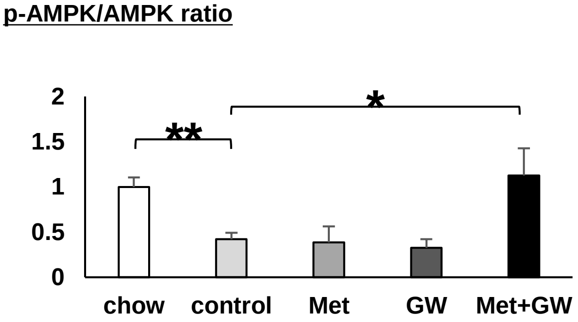

P-AMPK/AMPK ratios were expressed as fold change of chow (*p<0.05, **p<0.01).

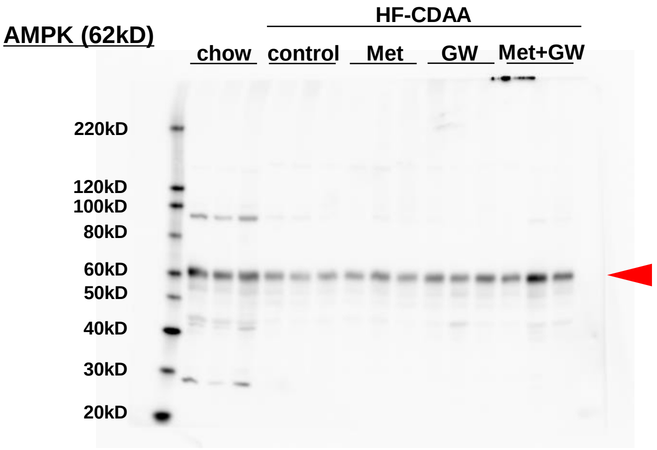

The blot was stripped and re-probed for AMPK (↑) and β-actin (↓).

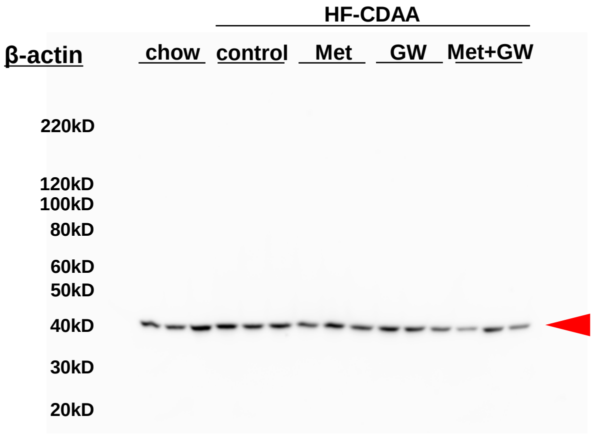

b **p-IRS-1 (Ser636/639), IRS-1 and β-actin of Figure 3E**

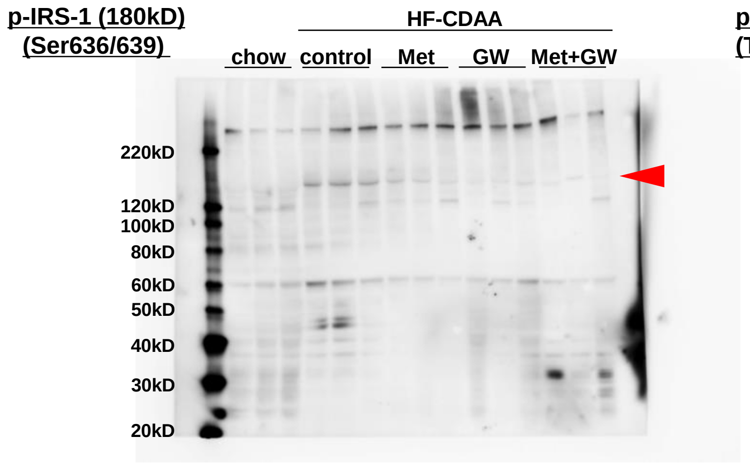

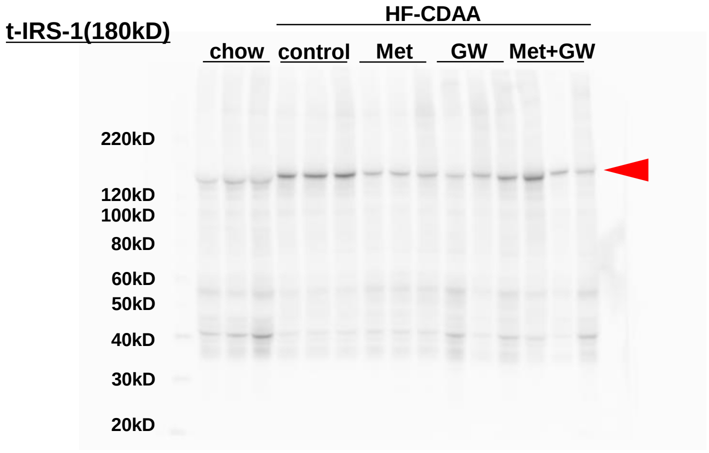

The blot was stripped and re-probed for IRS-1 (↑) and β-actin (↓).

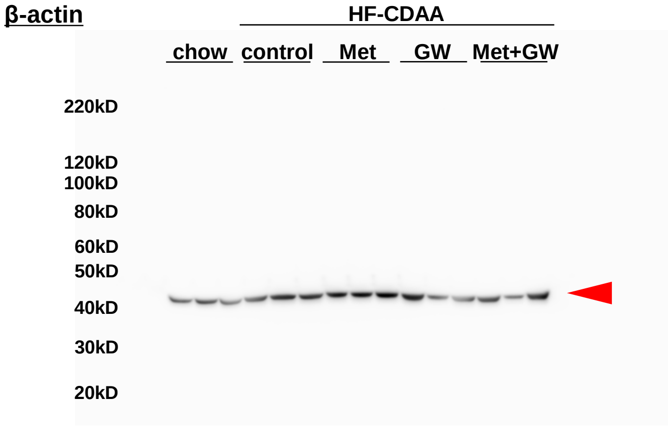

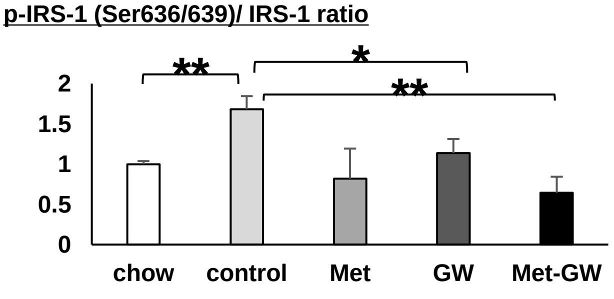

c **p-IRS-1 (Tyr896), IRS-1 and β-actin of Figure 3D**

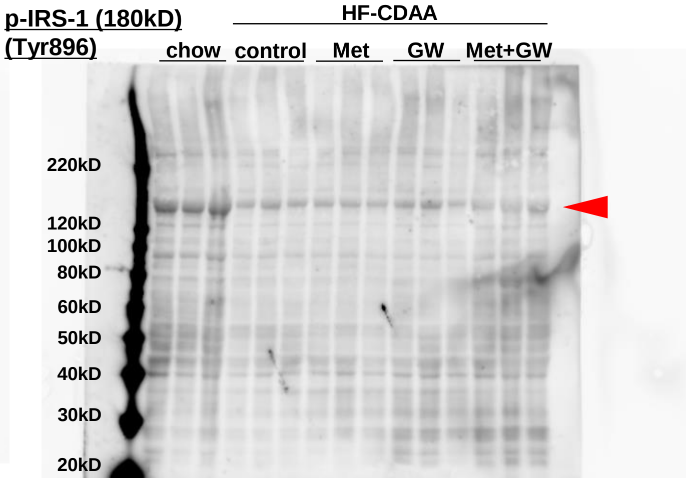

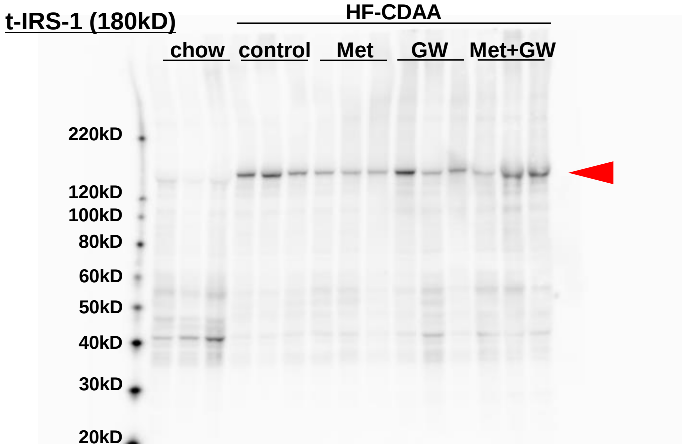

The blot was stripped and re-probed for IRS-1 (↑) and β-actin (↓).

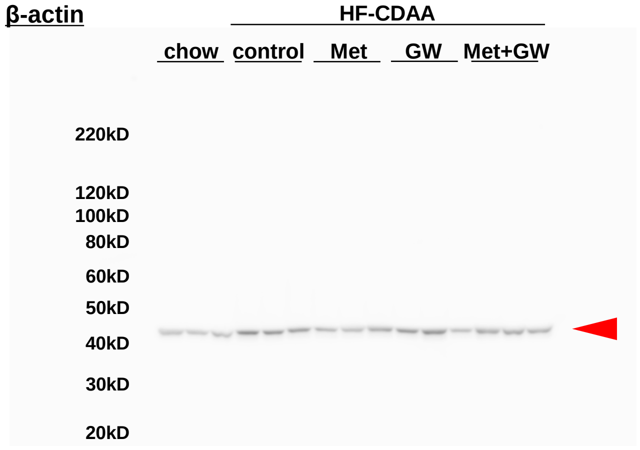

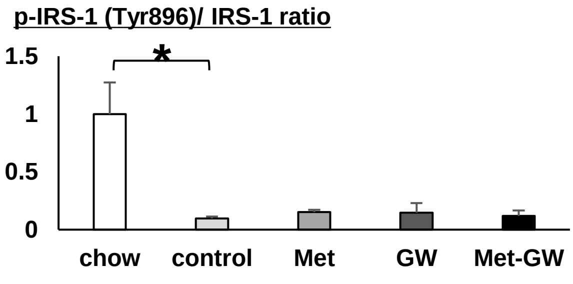

P- IRS-1 at ser636/639 and Tyr896/IRS-1 ratios in each group were expressed as fold change of chow (*p<0.05, **p<0.01).

Supplementary Figure S5. Uncropped images for which boxed areas are shown in the published panels J of Figure 1and panels E of Figure 3. (a) p-AMPK/ AMPK, (b) p-IRS-1 (Ser636/639)/ IRS-1 and (c) p-IRS-1 (Tyr896)/ IRS-1 levels were evaluated by immunoblot analysis of livers from 3 mice/group. Immunoblots of p-AMPK/ AMPK, p-IRS-1 (Ser636/639)/ IRS-1 and p-IRS-1 (Tyr896)/ IRS-1 were scanned, and band intensities were quantified by Image J (NIH) densitometry analysis. Each ratios were also expressed as fold change of controls. *$p<0.05$, **$p<0.01$.

a BiP and β-actin of Figure 4D

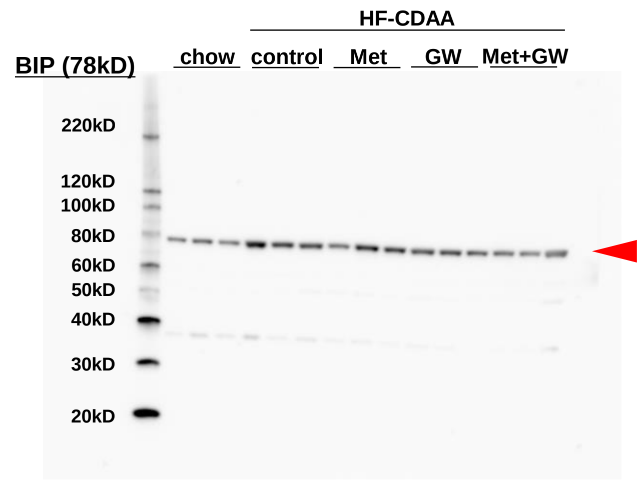

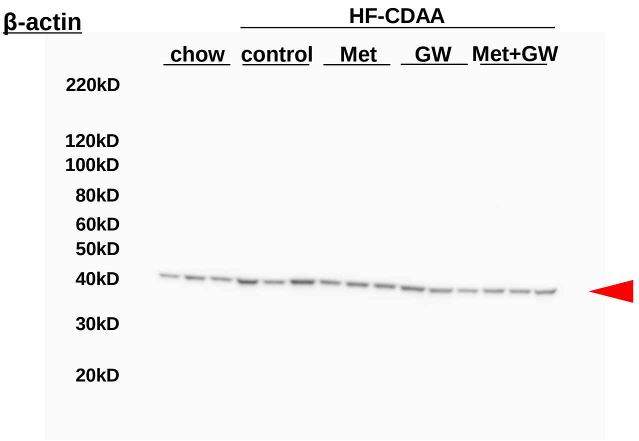

The blot was stripped and re-probed for β-actin (↑) .

b CHOP and β-actin of Figure 4D

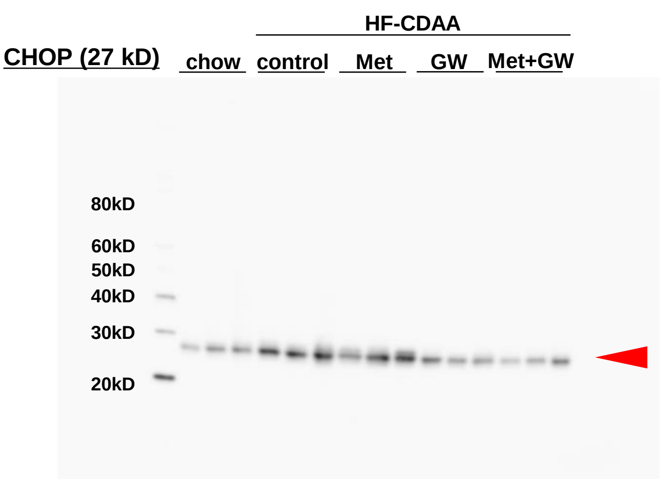

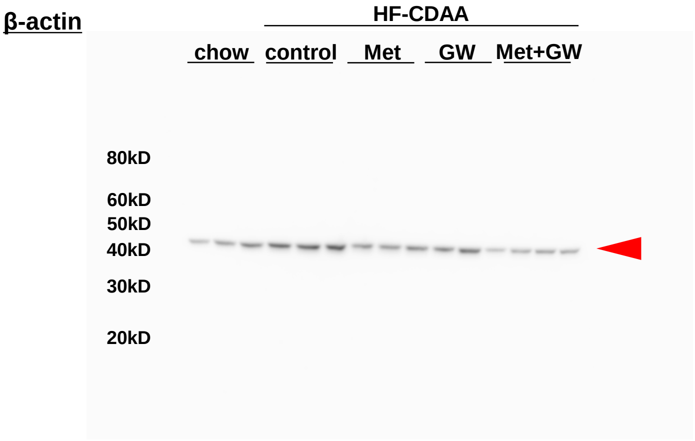

The blot was stripped and re-probed for β-actin (↑) .

BiP/ β-actin ratio

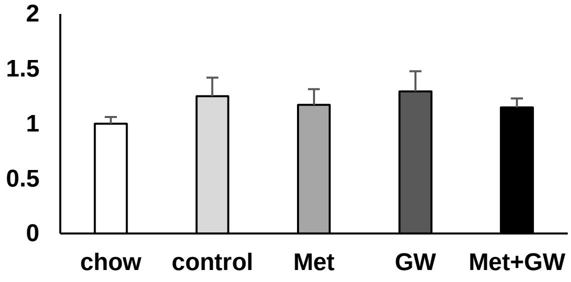

CHOP/ β-actin ratio

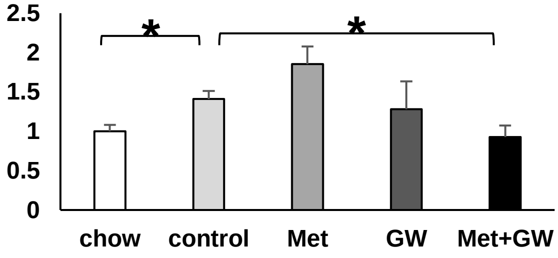

Bip/β-actin and CHOP/β-actin ratios were expressed as fold change of chow (*p<0.05).

C p-eIF2α, eIF2α and β-actin of Figure 4D

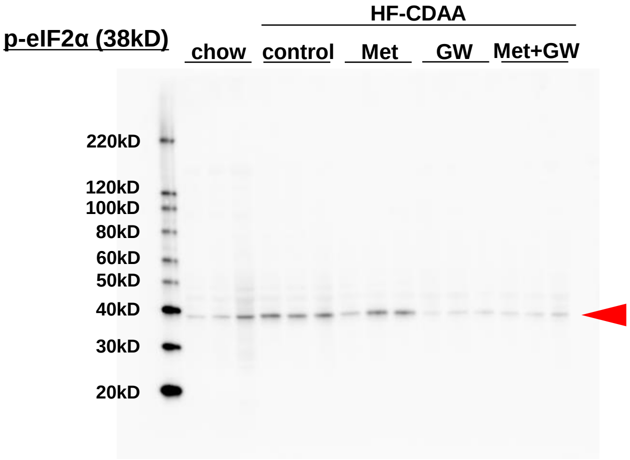

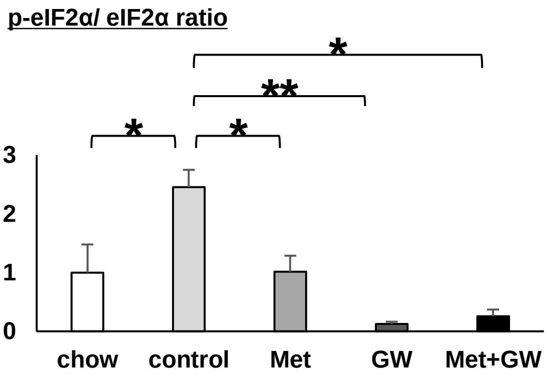

p-eIF2α/ eIF2α ratios were expressed as fold change of chow (*p<0.05, **p<0.01).

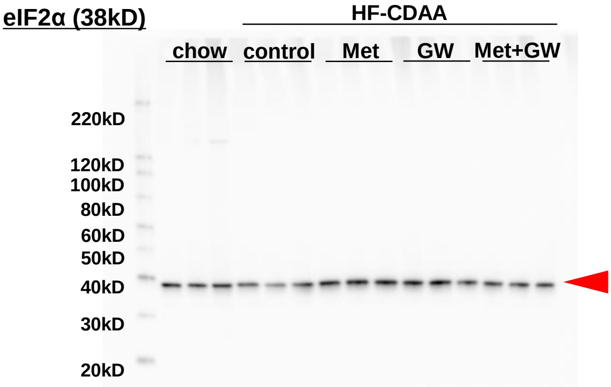

The blot was stripped and re-probed for eIF2α and then β-actin (↓).

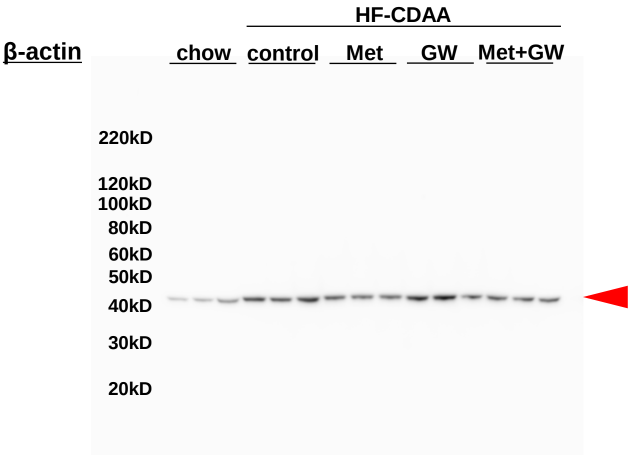

Supplementary Figure S6. Uncropped images for which boxed areas are shown in the published panels J of Figure 1and panels E of Figure 3. (a) BiP, (b) CHOP and (c) p-eIF2α/ eIF2α levels were evaluated by immunoblot analysis of livers from 3 mice/group. Immunoblots of BiP / β-actin
, CHOP/ β-actin and p-eIF2α/ eIF2α were scanned, and band intensities were quantified by Image J (NIH) densitometry analysis. Each ratios were also expressed as fold change of controls. *p<0.05, **p<0.01.
